# Redefining Red: Microbial Polyketides in Eco-Friendly Cosmetic Development

**DOI:** 10.1021/acsomega.5c10255

**Published:** 2025-12-08

**Authors:** Juliana Barone Teixeira, Pedro Garcia Pereira Silva, Júlio Gabriel Oliveira de Lima, Matilde Carvalho, Lídia M. Gonçalves, Nathalia Vieira Porphirio Veríssimo, Joana Marques Marto, Valéria C. Santos-Ebinuma

**Affiliations:** † Department of Bioprocess Engineering and Biotechnology, School of Pharmaceutical Sciences, 153998 São Paulo State University, Araraquara, SP 14800-903, Brazil; ‡ Research Institute for Medicines (iMed.ULisboa), Faculty of Pharmacy, 37809 Universidade de Lisboa, 1649-003 Lisbon, Portugal; § Department of Pharmaceutical Sciences, School of Pharmaceutical Sciences of Ribeirão Preto (FCFRP), University of São Paulo (USP), Ribeirão Preto 14040-020, Brazil

## Abstract

The increasing consumer demand for environmentally and health-conscious cosmetic products has propelled research into natural alternatives to synthetic colorants, which are often associated with negative health impacts. This study explores the development of facial cream, shampoo, and multifunctional jelly stick formulations incorporating lyophilized polyketide azaphilone red colorant produced by *Talaromyces amestolkiae*. The lyophilized extract was characterized by their antioxidant activity, cytotoxicity, and influence on the physicochemical properties of cosmetic formulations. At concentrations of 1 and 5 wt %, the extract demonstrated antioxidant capacity, reducing ROS levels by over 75% under oxidative stress while maintaining cell viability above 60%. Formulations containing the lyophilized extract exhibited suitable pH values, with rheological and texture analyses confirming their desirable application properties. Texture profile analysis indicated improved spreadability due to reduced adhesiveness and hardness, particularly at 1 wt % lyophilized extract concentration. Stability assessments revealed that color degradation was primarily influenced by temperature, with UV light having a lesser effect. The SWOT analysis identified microbial colorants as promising sustainable alternatives, despite current limitations in stability and regulatory hurdles. This study presents the potential of the lyophilized extract containing natural polyketide colorant produced through bioprocess as an eco-conscious alternative in cosmetics, offering both functional and environmental advantages over conventional synthetic dyes.

## Graphical abstract

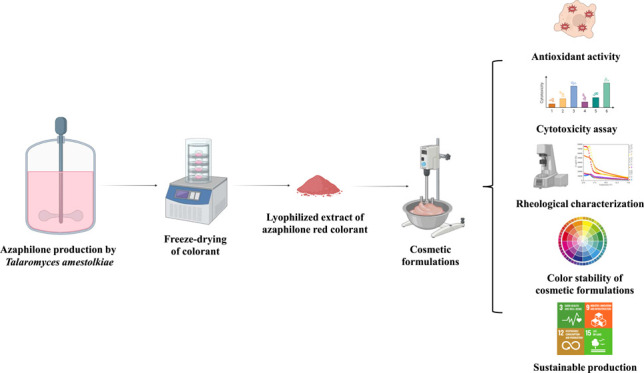

## 1 Introduction

Cosmetics have become an integral part of the population’s routine.^1^ Whether in the form of skincare and makeup or toiletries, consumers place great emphasis on wellness, fueling the demand for new and improved care products.^2^ By 2022, the cosmetics market was valued at USD 262.21 billion with a Compound Annual Growth Rate (CAGR) of 4.2% through 2030. However, only in 2023, the industry of skincare products was evaluated at approximately USD 625.7 billion, comprising a significant and rapidly expanding in the segment.^3^ Thus, growing consumer demand for sustainable and well-being-oriented products is driving the cosmetics industry toward natural and organic ingredients.^3,4^ This shift challenges manufacturers to maintain the high performance typically achieved by widely used synthetic substances like colorants and fragrances.^5^

Color is one of the leading contributors to product purchase, culminating in 60–90% of the perception of a product.^6^ Marketing strategies often use the physical attributes of products to evoke emotional responses to attract consumers.^7^ The additives used to give color to several products are the colorants which can be classified according to their source, structure and application method. Considering the colorant solubility, it is classified as dyes and pigments.^8,9^ In the cosmetic industry, it is quite common to find the term dye for the color additives. Moreover, exposure to colorants occurs mainly in areas close to mucous membranes.^10^ With the understanding that synthetic colorants dominate the market for color compounds, concerns over the health hazards attached to their uncontrolled use pushed for an expansion in the natural colorants market, evidenced by the estimated CAGR of 9.3% between 2022 and 2030.^11,12^

In this context, the filamentous fungus *Talaromyces amestolkiae* produces from yellow to red polyketide colorants with high industrial potential. These natural colorants are particularly valuable as alternatives to synthetic azo dyes, which provide vibrant color but are often toxic, contrary to the two prominent synthetic red dyes in the industry, Allura Red (Red 40) and Erythrosine (Red 3).^10^ Allura Red has been linked to allergies, behavioral disturbances, and genotoxicity, leading to its ban in several European countries.^13^ Similarly, Erythrosine has been associated with thyroid disorders, cancer, and DNA damage, prompting the Food and Drug Administration (FDA) to ban its use in food and ingested drugs after January 2025.^14^

Given these health concerns and potential future prohibitions, natural colorants from *T. amestolkiae* present a sustainable alternative. Their use aligns with the United Nations’ Sustainable Development Goals by promoting health, sustainable industrialization, responsible consumption, and ecosystem protection.^15^ Furthermore, the use of colorants of natural origins aligns with the marketability of products following consumers’ preferences for clean and health-conscious products.^11^

Hence, to fill the gap in the knowledge surrounding the application of natural colorants in cosmetic formulations, the present study proposes the physicochemical characterization (pH, particle size, rheology and texture analysis) and the evaluation of color stability over time in different conditions (presence and absence of light, and 40 °C) of shampoo, facial cream and multifunctional jelly stick formulations containing lyophilized extract containing a natural polyketide azaphilone red colorant produced by *T. amestolkiae*.

## 2 Material and Methods

### 2.1 Materials

Glyceryl stearate, cocamidopropyl betaine, sodium cocoamphoacetate, disodium PEG-5 laurylcitrate sulfosuccinate; sodium laureth sulfate, glycol distearate (and) steareth-4, polyglyceryl-3 dicitrate/stearate, diethylhexyl carbonate, PEG-7 glyceryl cocoate, PEG-120 methyl glucose dioleate and caprylic/capric triglyceride were purchased from EVONIK (Germany). Cetearyl alcohol was purchased from BASF (Portugal). Caprylyl/capryl glucoside and sodium cocoyl apple amino acids were purchased from SEPPC (France). Agar-agar was purchased from Iberagar (Portugal). Tocopherol was purchased from SM Nutritional Products (Switzerland).

### 2.2 Maintenance and Reactivation of Microorganism

The filamentous fungus *T. amestolkiae* DPUA 1275 was kindly provided by the Culture Collection of the Federal University of Amazonas (DPUA, AM, Brazil). The strain, originally preserved in distilled water, was reactivated following the procedure described by de Oliveira et al.,.^16^ Briefly, the culture was inoculated onto Petri dishes (90 mm × 10 mm) containing 10 mL of Potato Dextrose Agar (PDA) supplemented with 5 g·L^–1^ of yeast extract (YE) and incubated at 30 °C for 7 days.

### 2.3 Azaphilone Production by *T. amestolkiae*

Submerged production of colorants was carried out in a Minifors II bioreactor (Infors, New Jersey, USA) with a working volume of 4 L. The pre-inoculum was prepared by activating *T. amestolkiae* on Petri dishes (90 mm × 10 mm) containing 10 mL of Potato Dextrose Agar (PDA) supplemented with 5 g·L^–1^ of yeast extract (YE). A loopful of the preserved stock culture was streaked onto the medium and incubated at 30 °C for 7 days.

Following incubation, 45 agar plugs (8 mm diameter) containing active mycelium were aseptically custom-designed cutter and transferred to 500 mL Erlenmeyer flasks containing 100 mL of starter medium. The composition of the starter medium was (g·L^–1^): meat peptone (10), glucose (20), and meat extract (3). The flasks were incubated at 30 °C for 48 h in a rotary shaker (New Brunswick Innova 40R, Eppendorf, USA) at 150 rpm.

After cultivation, 400 mL of the resulting inoculum was aseptically transferred to the bioreactor containing 3.6 L of production medium under sterile conditions. The production medium consisted of (g·L^–1^): glucose (10), monosodium glutamate (25), MgSO_4_·7H_2_O (0.012), FeSO_4_·7H_2_O (0.010), and CaCl_2_ (0.015), adjusted to pH 5.0. Fermentation was conducted at 30 °C, 100 rpm, and pH 5.0 for 120 h.^16^

At the end of the cultivation period, the fermentation broth was clarified to obtain the colored fraction. The separation process involved vacuum filtration using 80 g·m^–2^ filter paper (Whatman, UK).

### 2.4 Freeze-Drying of Colorant

At the end of the fermentation process, the culture broth was subjected to solid–liquid separation as previously described. The resulting clarified supernatant, containing extracellular colorants, was stored at −80 °C for 24 h prior to freeze-drying.

The frozen supernatant was then lyophilized using a benchtop freeze-dryer (Modulyo D-230, Thermo Electron Corporation) under the following conditions: condenser temperature of −55 °C, chamber pressure of 0.1 mbar, and a total drying time of 48 h. All procedures were conducted under low-light conditions to minimize photodegradation of the colorant.

### 2.5 Assessment of Antioxidant Activity of Freeze-Dried Extract

The ability to reduce reactive oxygen species (ROS) of lyophilized extract was assessed using HaCaT cells.^17^ For this, HaCaT subconfluent cells grown in 96 well plates were incubated for 30 min with 20 μM of H_2_-DCFDA in the dark at 37 °C. Media was removed and fresh medium and added to the cells before being exposed to different concentrations of 1 mg mL^–1^ (C1) and 5 mg mL^–1^ (C5) of lyophilized extract and ascorbic acid (AA—vitamin C for positive control) for 1.5 h. Induction of ROS in cells was achieved by the addition of hydrogen peroxide (H_2_O_2_) solution (500 μM, 1 h) or exposure to UVB light (emission wavelength 32 nm) for 15 min. After the induction period the fluorescence was measured at 485 nm excitation and 520 nm emission wavelengths using fluorescence microplate reader (FLUOstar BMGLabtech, Germany). Analyses were carried out in 6 replicates and results were reported in percentage of ROS reduced as determined in eq 1.

1 $$ROS\;reduction\;\left(\%\right)=100-\left[\left(\frac{Fluorescence\;of\;exposed\;cells}{Fluorescence\;of\;unexposed\;control}\right)\times 100\right]$$

### 2.6 In Vitro Cytotoxicity Evaluation of Freeze-Dried Extract

Cytotoxicity was assessed using the end point MTT (3-(4,5-dimethyl-2-thiazolyl)-2,5-diphenyl-2H-tetrazolium bromide).^17^ Previously, cells HaCaT (CLS, Germany), were seeded in 96 well tissue culture plates at a cell density of 2 × 10^4^ cells per well, in RPMI 1640 culture medium supplemented with 10% fetal serum bovine, 100 units mL^–1^of penicillin G (sodium salt) and 100 μg mL^–1^ of streptomycin sulfate and 2 mM l-glutamine and incubated at 37 °C and 5% CO_2_ in a humidified atmosphere for 24 h. Then, cells were incubated with the freeze-dried extract at different concentrations (10, 5, 2.5, 1.3, 0.6, 0.3, 0.2, 0.1 mg mL^–1^ and a control −0 mg mL^–1^), culture medium and 1 mg mL^–1^ of sodium dodecyl sulfate (SDS) as negative and positive controls, respectively. After 24 h of exposition medium was replaced by a medium containing 0.5 mg mL^–1^ of MTT. After 3 h of incubation the formazan crystals were extracted with 100 μL of DMSO and the absorbance was measured at the wavelength of 570 nm in Microplate Reader (FLUOstar Omega, BMGLabtech, Germany). The relative cell viability was determined by eq 2.

2 $$Relative\;cell\;viability\;\left(\%\right)=\frac{Sample\;absorbance}{Negative\;Control\;absorbance}\times 100$$

### 2.7 Development of Cosmetic Formulation

Cosmetic formulations were developed by incorporating the lyophilized extract containing natural polyketide azaphilone red colorant at concentrations of 1 and 5 wt %. These formulations were used to produce three different cosmetic products: a facial cream, a shampoo, and a jelly multifunctional stick. The following section details the preparation and characterization of these formulations.

#### 2.7.1 Formulation of Facial Cream

Facial creams were prepared by adding the oily and nonoily compounds to separate stainless-steel bowls, which were then placed in a water bath at 85 °C and stirred for 5 ± 1 min to ensure complete homogenization.^4^ All ingredients used in facial cream formulation are presented in Table S1. Afterward, the oily ingredients were gradually added to the bowl containing the nonoily ingredients, and the mix was manually stirred until cold. The azaphilone extract was then added and the emulsion was stirred until homogeneous.

#### 2.7.2 Formulation of Shampoo

Shampoo formulations were modified from Nunes et al.,^18^ and prepared by adding all ingredients to a stainless-steel bowl and placing them in a water bath at 85 °C until total homogenization. All ingredients used in shampoo formulation are presented in Table S2. Subsequently, the mix was set aside until cold. The azaphilone extract was added and the formulation was stirred for 5 ± 1 min to ensure complete homogenization.

#### 2.7.3 Formulation of Jelly Multifunctional Stick

Jelly multifunctional sticks were prepared by adding all ingredients to a stainless-steel bowl and placing them in a water bath at 85 °C until total homogenization. All ingredients used in the jelly multifunctional stick formulation are presented in Table S3. Afterward, while the mix was still liquid, the azaphilone extract was added and stirred until homogeneous.

### 2.8 Physicochemical Characterization of Cosmetic Formulation

Each cosmetic formulation produced with the natural colorant from *T. amestolkiae* presents specific characteristics due to differences in composition and intended application. Therefore, the formulations were analyzed individually, and their physicochemical properties are presented below.

#### 2.8.1 Measurement of pH

The pH of the facial cream and shampoo formulations was measured at room temperature 24 h after preparation, using an EutechTM Ion 2700 m pH meter (Thermo Scientific, Massachusetts, USA) previously calibrated.^5^

#### 2.8.2 Rheological Characterization

Rheological analyses were performed on the facial cream and shampoo formulations using a controlled stress Malvern Kinexus Lab + Rheometer (Malvern Instruments, Malvern, UK) using cone-and-plate geometry (truncated angle 4° and radius 40 mm). The shear rate method was applied to determine the dynamic viscosity of the formulations. Shear stress was obtained by increasing the shear rate from 0.1 to 100 s^–1^. Oscillation frequency sweep tests were performed at frequencies ranging from 0.1 to 100 Hz, with a shear rate of 1%. All tests were performed at 25 °C, 24 h after preparing the formulations.^5^

Oscillatory tests were carried out to analyze viscoelastic properties at different temperatures and to determine the ideal working and solidification temperature for jelly multifunctional stick formulations in a controlled stress Malvern Kinexus Lab + Rheometer (Malvern Instruments, Malvern, UK). Thus, the temperature varied from 25 to 75 °C at a rate of 2.5 °C.min^–1^, with a frequency fixed at 1 Hz and a shear stress of 1%.

#### 2.8.3 Texture Profile Analysis (TPA)

The formulations of facial creams and shampoos were characterized according to their texture profile using the TA.XTplusC texture analyzer (Stable Microsystems, Godalming Surrey, UK), equipped with a 10 kg load cell, applying a cylindrical probe with a diameter of 0.5 in and a test speed of 5 mm s^–1^. Parameters calculated through the TPA were hardness, adhesiveness, elasticity and cohesiveness.^19^ For the jelly multifunctional stick, a hardness assay was performed using a TA.XTplusC texture analyzer (Stable Microsystems, Godalming Surrey, UK), using a 2 mm diameter cylindrical probe, a test speed of 0.5 mm s^–1^, and a trigger force of 0.010 N.^20^

#### 2.8.4 Droplet Size Distribution

Droplet size distribution was obtained for facial cream formulations with 1 wt % *T. amestolkiae* extract using the Malvern Mastersizer 2000 equipment (Malvern Instruments, Worcestershire, UK) coupled with a Hydro S accessory. Approximately 0.5 g of the formulation was added to 120–150 mL of water in the sample chamber, with obscurity between 10 and 20%, and agitation at 1750 rpm. The data was expressed in terms of relative volume distribution and given as diameter values corresponding to percentiles of 10, 50 and 90% (mean ± SD, *n* = 6). The tests were performed 48 h after preparing the formulations.^4^

### 2.9 Evaluation of Color and pH Stability in the Developed Formulations

The stability of all three formulations was analyzed according to their color properties, using a MetaVueTM colorimeter (X-Rite, Michigan, USA), and pH, using a SevenEasyTM pH meter (Mettler Toledo, Greifensee, Switzerland).^21^

The facial cream, shampoo, and multifunctional stick formulations, which contained 1 wt % of azaphilone extract from *T. amestolkiae*, were kept under three different conditions: (a) 25 °C in the absence of light, (b) 25 °C under sunlight exposure, and (c) 40 °C in the absence of light. The analysis was performed at 0 h (control), 7, 15, and 30 days.

### 2.10 Statistical Analysis

Statistical analysis was performed using Excel Software. For antioxidant activity and in vitro cytotoxicity assays, one-way ANOVA was performed, followed by multiple comparisons using Tukey’s test. Differences were considered significant for *p* < 0.05.

### 2.11 SWOT Analysis of Natural Colorants Applied to Cosmetic Formulations

To evaluate the potential of natural red colorants as alternatives in cosmetic formulations, a SWOT (Strengths, Weaknesses, Opportunities, Threats) analysis was conducted. The methodology was adapted from Ram at al.,^22^ and the analysis categorized factors into: Strengths (S) and Weaknesses (W): Internal, controllable attributes of natural red colorants. Opportunities (O) and Threats (T): External, noncontrollable factors influencing their adoption.

## 3 Results and Discussion

### 3.1 Natural Red Colorant as an Alternative to Synthetic Red Dyes

In this study, cosmetic formulations were developed using lyophilized extract containing a natural polyketide azaphilone red colorant obtained through *T. amestolkiae* cultivation, offering a sustainable alternative to synthetic colorants. In this scenario, microbial colorants such as azaphilones, present a potential eco-friendly colorant solution that aligns with global efforts to reduce reliance on petrochemical-derived colorants.^23,24^ By incorporating this natural colorant at concentrations of 1 and 5 wt %, three cosmetic products—a facial cream, a shampoo, and a multifunctional stick—were formulated. This approach not only enhances the environmental profile of the products but also supports the SDGs. The following discussion evaluates the performance, stability, and potential of these formulations with the natural azaphilone colorant as sustainable alternatives to the ones with synthetic counterparts.

#### 3.1.1 Antioxidant Activity and Cytotoxicity Assays from Lyophilized Extract

The antioxidant activity of the fungal lyophilized extract solution was analyzed using the HaCaT method with H_2_-DCFDA for quantification. H_2_-DCFDA is a nonfluorescent molecule that is hydrolyzed by intracellular enzymes to the nonfluorescent 2′-7′-dichlorodihydrofluorescein (H_2_DCF), which reacts with ROS, resulting in DCF, a fluorescent compound.^17^ As shown in Figure 1A,B, C1 (1 mg mL^–1^) of lyophilized extract, the percentage of ROS reduction in cells exposed to UVB light or H_2_O_2_ was significantly lower than the percentage obtained with ascorbic acid. However, this same concentration was able to reduce ROS formation by more than 75% in both oxidative stress conditions, thus demonstrating high antioxidant activity in the HaCaT cell line exposed to oxidative stress. In C5 (5 mg mL^–1^) concentration of freeze-dried *T. amestolkiae* polyketides azaphilones, the analyses of ROS reduction were deemed inconclusive as the samples at higher concentrations exhibited fluorescence at the same wavelength used for the assays. Therefore, results obtained with UVB induction could be underestimated, meanwhile, results for reduction after H_2_O_2_ induction were not possible due to the interference.

**Figure 1:**
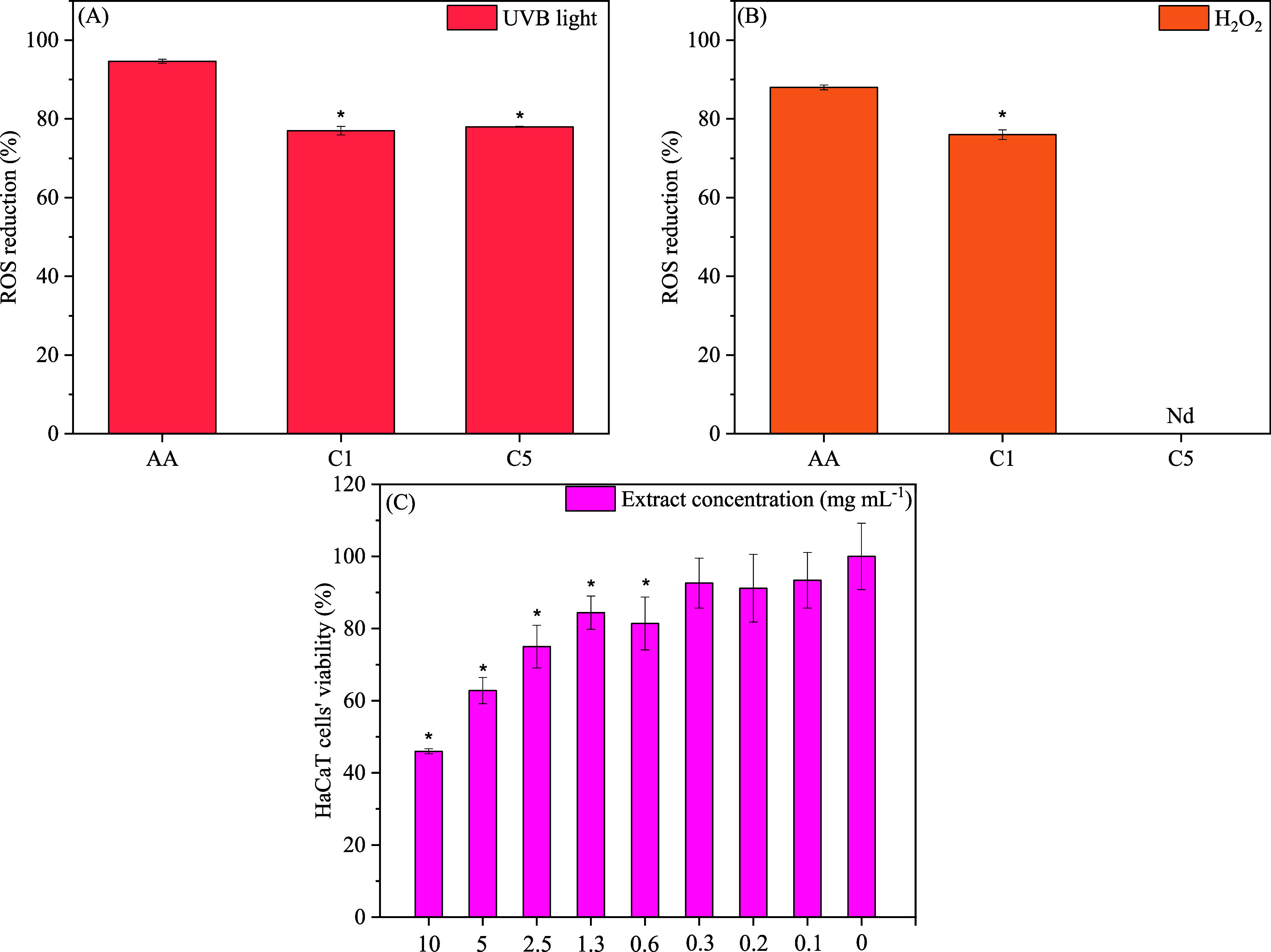
Assessment of antioxidant activity and in vitro cytotoxicity of lyophilized extracts of *T. amestolkiae* *.* (A) Antioxidant activity under UVB light exposure; (B) Antioxidant activity under H_2_O_2_ exposure; (C) In vitro cytotoxicity evaluation under different concentrations of colored extracts. AA—ascorbic acid (positive control); C1 and C5—1 and 5 mg mL^–1^ of lyophilized extract. * Different from control (*p* < 0.05). Nd = Not determined.

The cytotoxicity assays of lyophilized extract were performed on HaCaT cells and evaluated using the MTT reduction method. The results presented in Figure 1C showed that cell viability decreases with increasing sample concentration. Nevertheless, cell cultures exposed to polyketide azaphilones at concentrations of ≤5 mg mL^–1^ exhibit viabilities above 60% which, together with the results of IC_50_ (concentration of extract needed to reduce the cell viability to 50%) of 7.6 ± 0.9 mg mL^–1^, suggest that the samples are suitable for topical application.

These conclusions are in agreement with those presented by Zaccarim et al.,.^25^ The cell viability of *T. amestolkiae* azaphilones extract was evaluated in fibroblasts, resulting in an IC_50_ of 187.5 mg mL^–1^. This concentration was higher than the one obtained in this study, however, corroborates the conclusion that the extract in question has low cytotoxicity.

### 3.2 Physicochemical Characterization of Cosmetic Formulation

In cosmetic development, pH is an important factor to be considered, as using products with inadequate pH values can cause sensibility, dryness, and weakening of the skin barrier and hair.^26^ The effect of lyophilized *T. amestolkiae* polyketides azaphilones extract in the pH of facial cream and shampoo formulations was evaluated in 1 and 5 wt % concentrations in Table 1. The ideal pH for facial cosmetics ranges between 4.1 and 5.8.^27^ Thus, all formulations of facial cream, with and without natural colorant, were adequate. The pH for shampoos should not exceed 5.5, therefore, the addition of azaphilones polyketides was seen to have improved the formulations as it reduced the pH from 5.93 ± 0.42 of control formulation, to 5.06 ± 0.03 and 5.10 ± 0.01 of 1 and 5 wt % formulations, respectively, allowing them to fit within the acceptable range.^26^

**Table 1:** Values of pH for Facial Cream and Shampoo Formulations, With and Without the Addition of Lyophilized Extract

formulation	colorant lyophilized extract (wt %)	pH
facial cream	–	5.17 ± 0.30
	1	5.24 ± 0.36
	5	5.27 ± 0.36
shampoo	–	5.93 ± 0.42
	1	5.06 ± 0.03
	5	5.10 ± 0.01

### 3.3 Rheological Characterization in the Developed Formulations

Rheology characterization is used to assess product structures and mechanical properties that affect product spreadability, lubricity and performance. In shampoo formulations, the control showed higher viscosity values of 0.484 Pa.s at 0.1 s^–1^, although not considerably different from formulations with 1 and 5 wt % freeze-dried extract which showed viscosity values of 0.237 and 0.459 Pa.s, respectively. Additionally, the flow curve presented in Figure 2A shows that the viscosity is constant with the increase of shear strain, which reflects the linear increase in shear stress with the increase of shear strain, characterizing the shampoo formulations as Newtonian fluids.^18^

**Figure 2:**
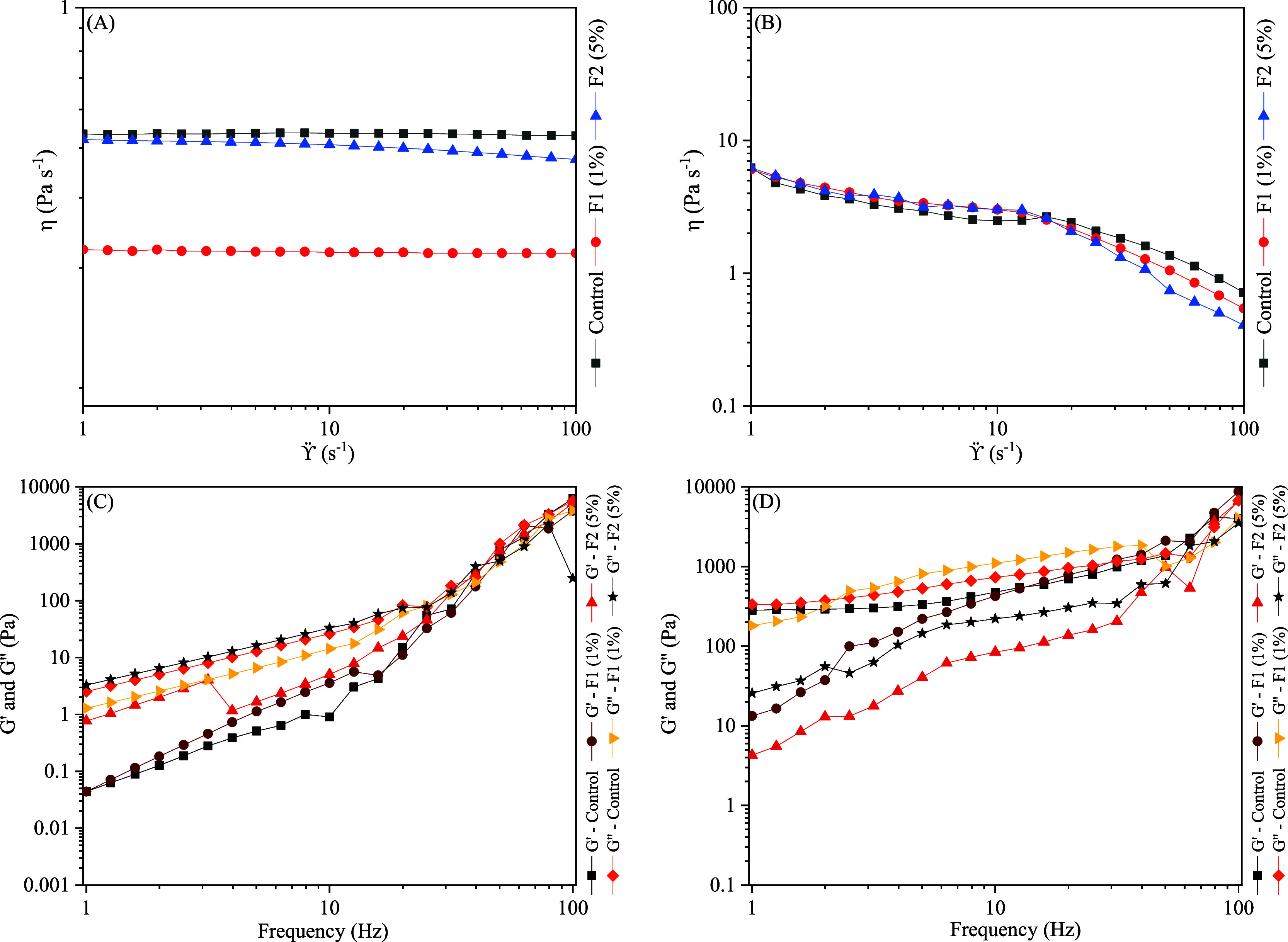
Rheological characterization in the developed formulations. The relationship between shear rate and viscosity for cosmetic formulations with added colorant: (A) shampoo; (B) face cream. Relationship between frequency G′ and G″ for cosmetic formulations with added colorant. (C) Shampoo; (D) facial cream.

In facial creams, flow curves show that the apparent viscosity decreases with increasing shear rate, an indication of shear-thinning behavior, as presented in Figure 2B. In formulations with topic application, such as facial creams, shear-thinning is a desirable behavior, as it allows for the formulation to easily and evenly spread.^28^ Figure S1 correlates to the results observed, as the flow curves, in which the shear strain increased and decreased from 0.1 to 100 s^–1^ in 10 min, indicate the presence of thixotropy. This property is only present in non-Newtonian fluids and is used to characterize the reversibility of time-dependent structural change in materials. In cosmetic emulsions, the viscosity of a formulation should decrease at a high shear rate (shear thinning properties) to cover the skin easily and evenly. However, it should be recovered fast enough after application to form a protective barrier on the skin. Thus, the presence of thixotropic behavior is commonly observed in topical creams.^17,29^

The oscillatory studies indicate the system’s response as a function of frequency at constant shear strain, providing information on the storage modulus or elastic component (G′) and the loss modulus or viscous component (G″). As shown in Figure 2C–D, in both formulations, the flow curves indicate that the addition of polyketides azaphilones did not cause significant changes in the viscoelasticity. Initially, G″ was superior to G′, indicating a predominance of viscous effects, however, at around 40 Hz, storage modulus and loss modulus intersect, pointing to the transition from a liquid-dominated state to a solid-dominated state.

In shampoos, G″ > G′ is to be expected, considering the low viscosity levels of the formulations and Newtonian fluids properties. Additionally, this indicates the products can be easily applied to hair washing.^18^ The results obtained for facial creams indicated that the formulations developed presented a more liquid-like structure than those reported in the literature, meaning they are less rigid and can be more easily applied.^4^

Similar to lipsticks, when formulating multifunctional sticks determining the behavior of the formulation as a function of temperature is crucial to understanding the applicability and performance of the product. The temperature ramp presented in Figure 3 indicates that the jelly formulation exhibits that the storage modulus overcomes the loss modulus (G′ > G″) regardless of the working temperature, indicating a solid-like behavior of the formulation. Additionally, this profile allows the determination of the complete solidification temperature of the multifunctional stick at 30 °C, which is desired when working with these kinds of cosmetics as they should stay firmly solid in ambient conditions.

**Figure 3:**
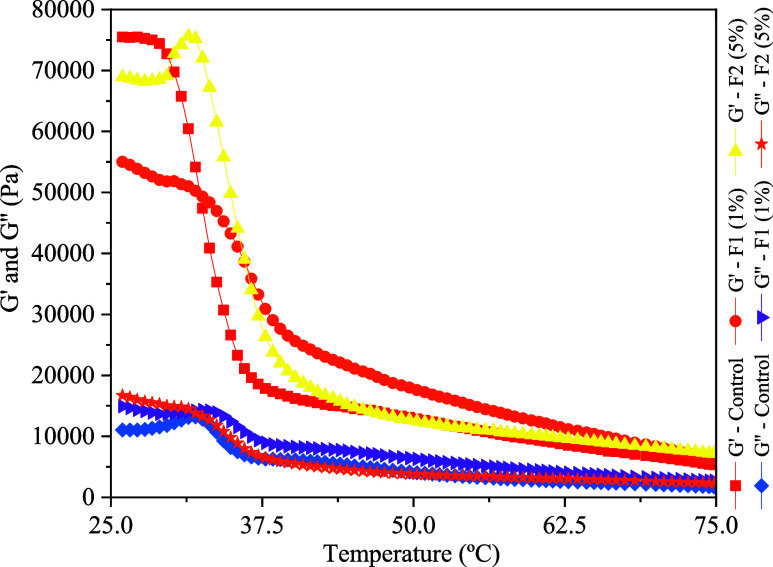
Loss and elastic modulus variation with temperature of multifunctional jelly stick formulations with 1 and 5 wt % of lyophilized extract.

Thus, the rheology assays indicated that the use of colorant lyophilized extract colorant did not significantly alter the rheological properties of facial cream, shampoo and multifunctional jelly stick formulations, suggesting that the performance of these cosmetics would remain satisfactory with the addition of this natural colorant.

### 3.4 Texture Profile Analysis (TPA)

TPA is a popular method to study different properties of cosmetic formulations and was performed in shampoo and facial cream formulations. As shown in Table 2, hardness values for 1 wt % of polyketide azaphilones were similar to those obtained for the control formulation, while 5 wt % of polyketide azaphilones showed slightly higher results. Cohesiveness is related to the ability to resist deformation before rupture. In shampoos and facial creams, cohesiveness was the highest in 5 wt % of polyketide azaphilones in 25 and 36%, respectively. Hardness and cohesiveness values were generally lower than those reported in literature, for shampoos and moisturizing creams, which should be associated with how the cosmetics developed in this study were formulated to be less thick and more easily absorbed.^30,31^

**Table 2:** TPA Evaluation of Shampoo and Facial Cream Formulations, With and Without the Addition of Lyophilized Extract

formulation	colorant lyophilized extract (wt %)	hardness (g)	adhesiveness (g.s)	elasticity	cohesiveness
shampoo	–	3.906 ± 0.056	–3.392 ± 0.331	0.879 ± 0.242	–0.194 ± 0.008
	1	3.727 ± 0.127	–3.578 ± 0.717	1.165 ± 0.025	–0.243 ± 0.104
	5	4.107 ± 0.137	–9.035 ± 0.840	1.222 ± 0.105	–0.005 ± 0.013
facial cream	–	3.887 ± 0.231	–7.079 ± 0.314	1.345 ± 0.105	0.042 ± 0.005
	1	3.976 ± 0.033	–9.251 ± 0.940	1.348 ± 0.009	0.03 ± 0.002
	5	4.125 ± 0.137	–20.97 ± 0.084	1.497 ± 0.021	0.116 ± 0.037

Adhesiveness is a parameter that determines the energy required to overcome the attraction between the surface of the material and another surface. The addition of polyketide azaphilones in shampoos resulted in 1.05- and 2.66-times lower adhesiveness, for 1 and 5 wt % of polyketide azaphilones, respectively. Meanwhile, in facial creams 1 and 5 wt % of polyketide azaphilones resulted in 1.31 and 2.96 lower adhesiveness. Cohesiveness and adhesiveness are directly linked to the spreadability of cosmetics. Lower values of these properties, while still exhibiting a balance, are desirable for better application.^32^ Therefore, the addition of polyketide azaphilones was beneficial for shampoos and facial creams, in particular for the lower values of adhesiveness, as it assures a nonsticky and nonstiff formulation.

Jelly multifunctional sticks are a hydrophilic alternative to wax-based sticks, such as traditional lipstick formulations. Although jelly sticks and lipsticks present similar physical attributes and can be used for the same purpose, of applying pigment and dyes to the lips, and if desired to the cheek and eye area, their rheological and textural characteristics can vary as jelly sticks can maintain their structure and be easily applied, all while having a less rigid formulation, due to the presence of water as its main component.

Thus, analyzing hardness in innovative formulations, such as the jelly sticks in this study, is necessary to evaluate if the product is applicable without damaging itself or the skin, and to determine if it can be applied for what is intended. In conventional stick formulations, hardness should be above 40 g, to maintain integrity during application and storage, however should not be too high to make the application of the product unsatisfactory.^20^ Table 3 shows that all of the formulated multifunctional jelly sticks, with and without the addition of 1 and 5 wt % of lyophilized extract, resulted in lower values of hardness than the control. While formulation containing 1 wt % reduced the value of hardness to an acceptable for the performance of the multifunctional stick (62.462 ± 17.598 g), the addition of the polyketide azaphilones in higher concentration reduced the hardness to a value outside of the ideal range previously mentioned (23.296 ± 17.602 g). Therefore, the presence of 1 wt % of the extract reduced the hardness results to an optimized value when compared to the control formulation, without compromising the structure of the product.

**Table 3:** Hardness Evaluation of Jelly Multifunctional Stick With and Without Colorant Lyophilized Extract (Mean ± SD, *n* = 6)

colorant lyophilized extract (wt %)	hardness (g.s)	absolute positive force (g)
–	0.0075 ± 0.0005	100.935 ± 43.375
1	0.0092 ± 0.0003	62.462 ± 17.598
5	0.0071 ± 0.0005	23.296 ± 17.602

Rheological and textural characterization revealed that formulations containing 1 and 5 wt % of lyophilized extract exhibited comparable behavioral profiles across all cosmetics formulations developed. Given the equivalent performance and the sustainable advantage of reducing raw material costs, the 1 wt % concentration was selected for subsequent stability evaluation. This approach not only ensures cost-effective production but also aligns with sustainable formulation practices by minimizing resource use without compromising product quality.

### 3.5 Characterization of the Particle Size Distribution

The characterization was conducted to evaluate the influence of incorporating polyketide azaphilones on the particle sizes of the emulsions as presented in Table 4. The formulation with a concentration of 1 wt % of lyophilized extract, has 50% of its particles smaller than 7.453 ± 0.106 μm, while the control formulation (without the addition of extract) has 50% of particles smaller than 10.445 ± 0.114 μm. This correlated to the results obtained in the rheology assays, as emulsions with larger particle sizes exhibit higher viscosity.^5^ However, despite presenting a smaller average, emulsions with 1 wt % lyophilized extract have much larger particles in the 90th percentile (52.592 ± 0.359 μm), which indicates a greater proportion of very large particles. This statement is proven by the Span results obtained.

**Table 4:** Droplet Size for Facial Cream Formulation With and Without the Addition of Lyophilized Extract (Mean ± SD, *n* = 6)

droplet size distribution (μm)				
sample	span	d (10)	d (50)	d (90)
control	1.985 ± 0.010	1.983 ± 0.010	10.445 ± 0.114	22.710 ± 0.154
1 wt %	6.833 ± 0.191	1.614 ± 0.007	7.453 ± 0.106	52.592 ± 0.359

The control sample presents a reduced Span value (1.985 ± 0.010 μm) when compared to the sample of emulsions with 1 wt % of polyketide extract (6.833 ± 0.191 μm), this indicates that the formulations without the addition of colorants obtained less variation in particle size, while the addition of lyophilized extract caused greater heterogeneity. Less cohesive formulations tend to show less homogeneity, therefore, the results shown in this study are in agreement with those established by the texture profile analysis, as cohesiveness was lower for facial creams with lyophilized extract.^17^ Thus, the average particle size in emulsions with and without the addition of lyophilized extract was not so discrepant and complies with values reported in the literature, which can vary from 0.1 and 50 μm for formulations of this type.^18^

### 3.6 Stability of Cosmetic Formulations

One of the main challenges in using natural colorants is their low stability under stress conditions (for example, exposure to light and high temperatures).^33^ Therefore, evaluating the color stability of these formulations was crucial to understanding the fragility of polyketide azaphilone molecules when applied in cosmetics formulations.

Based on the Δ*E* values shown in Figure 4, it can be observed that after 7 days—both with and without light exposure—the facial cream exhibited no perceptible color changes (Δ*E* < 2). However, the effect of temperature was notable even within this 1 week, with Δ*E* slightly exceeding 4. Furthermore, after 15 days of testing, the formulation displayed significant color variation under all tested conditions. Notably, the control formulation, subjected to the same conditions, showed Δ*E* variations between 1.5 and 1.7. This suggests that the color changes observed in the colored facial cream may be partially attributed to alterations in the base formulation itself.

**Figure 4:**
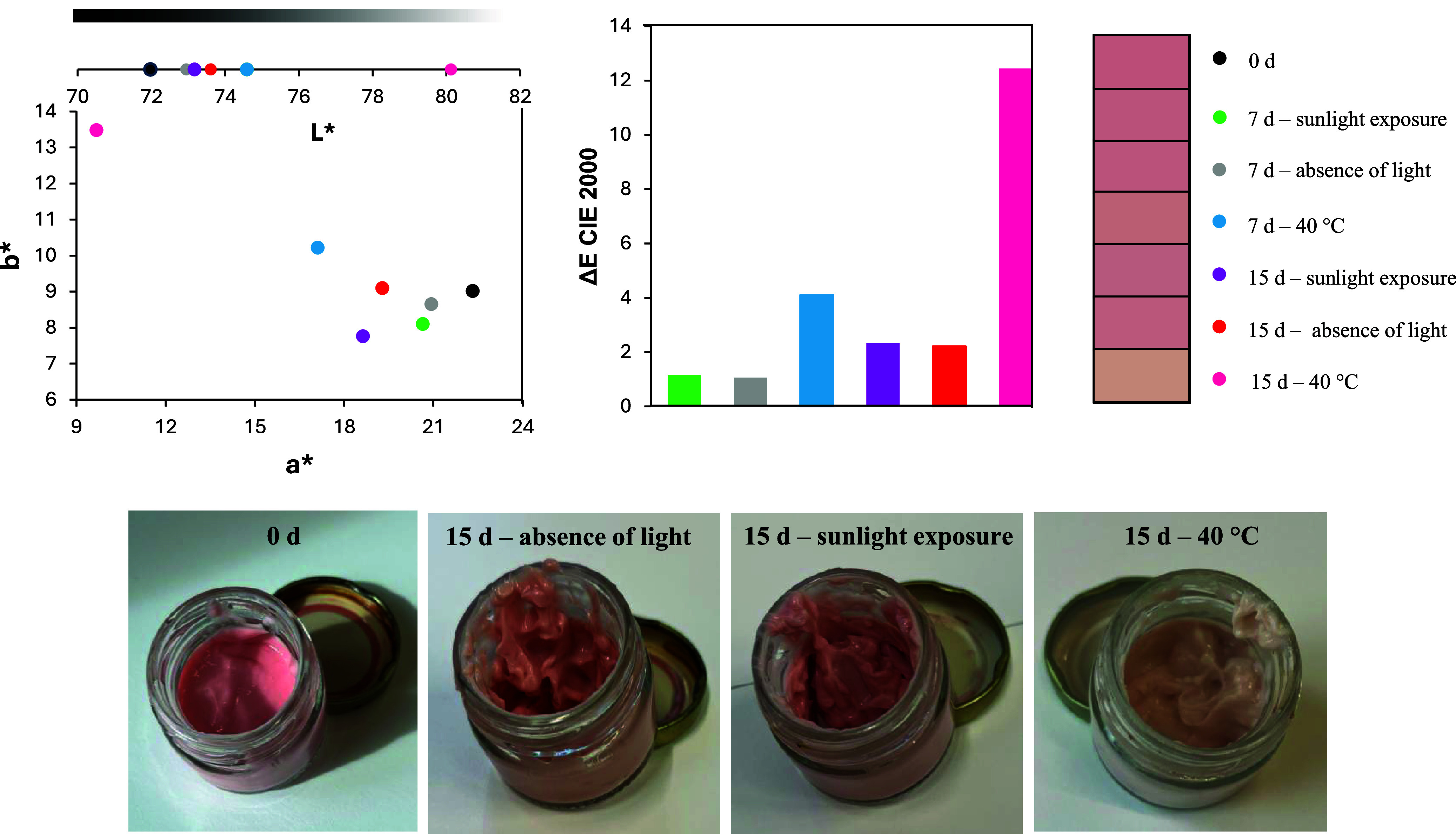
Color properties in the CIELAB system of facial cream formulation with 1 wt % of lyophilized extract under different storage conditions over time.

In shampoo formulations as presented in Figure 5, a significant color difference (Δ*E*) was observed across all tested time points and conditions. The control formulations also exhibited notable color variation (Δ*E* > 2), suggesting that the observed changes in azaphilone polyketide-containing formulations may be partly linked to inherent instability in the base formulation. Additionally, the presence of pearlescent agents likely contributed to shifts in the L* (lightness) parameter, which showed the most pronounced variation among all color parameters. Under oven conditions (40 °C), the shampoos displayed a distinct behavior: the b* parameter increased significantly, leading to a more yellowish hue. This phenomenon may be attributed to the lower stability of red chromophores, which degraded first, allowing the yellow-orange chromophores to dominate.^11^

**Figure 5:**
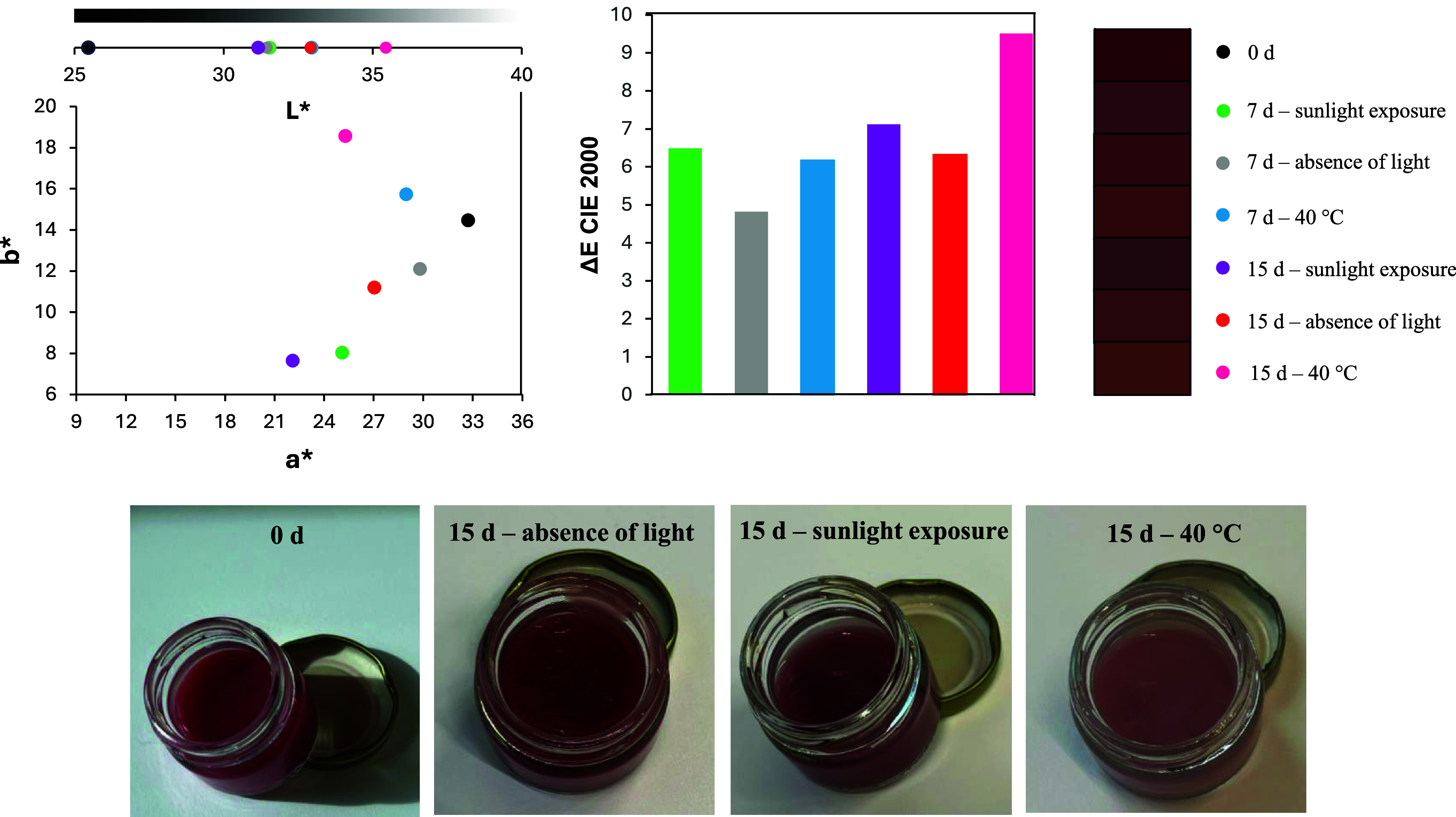
Color properties in the CIELAB system of shampoo formulation with 1 wt % of lyophilized extract under different storage conditions over time.

Concerning the color stability of multifunctional lipstick as presented in Figure 6, a significant color difference (Δ*E*) was observed across all tested periods and conditions. The control formulations also showed notable color variation (Δ*E* > 2), suggesting these changes may be associated with the inherent instability of azaphilone polyketide-containing formulations. The Δ*E* plot further revealed that elevated temperature (40 °C) had a more pronounced impact on chromophore degradation than light exposure. Specifically, the color variation after just 7 days at 40 °C was comparable to that observed after 15 days under light exposure. Notably, while both conditions caused color changes, only thermal degradation resulted in a progressive yellowing of the formulation. This distinct behavior implies that storage conditions differentially affect chromophore degradation pathways.

**Figure 6:**
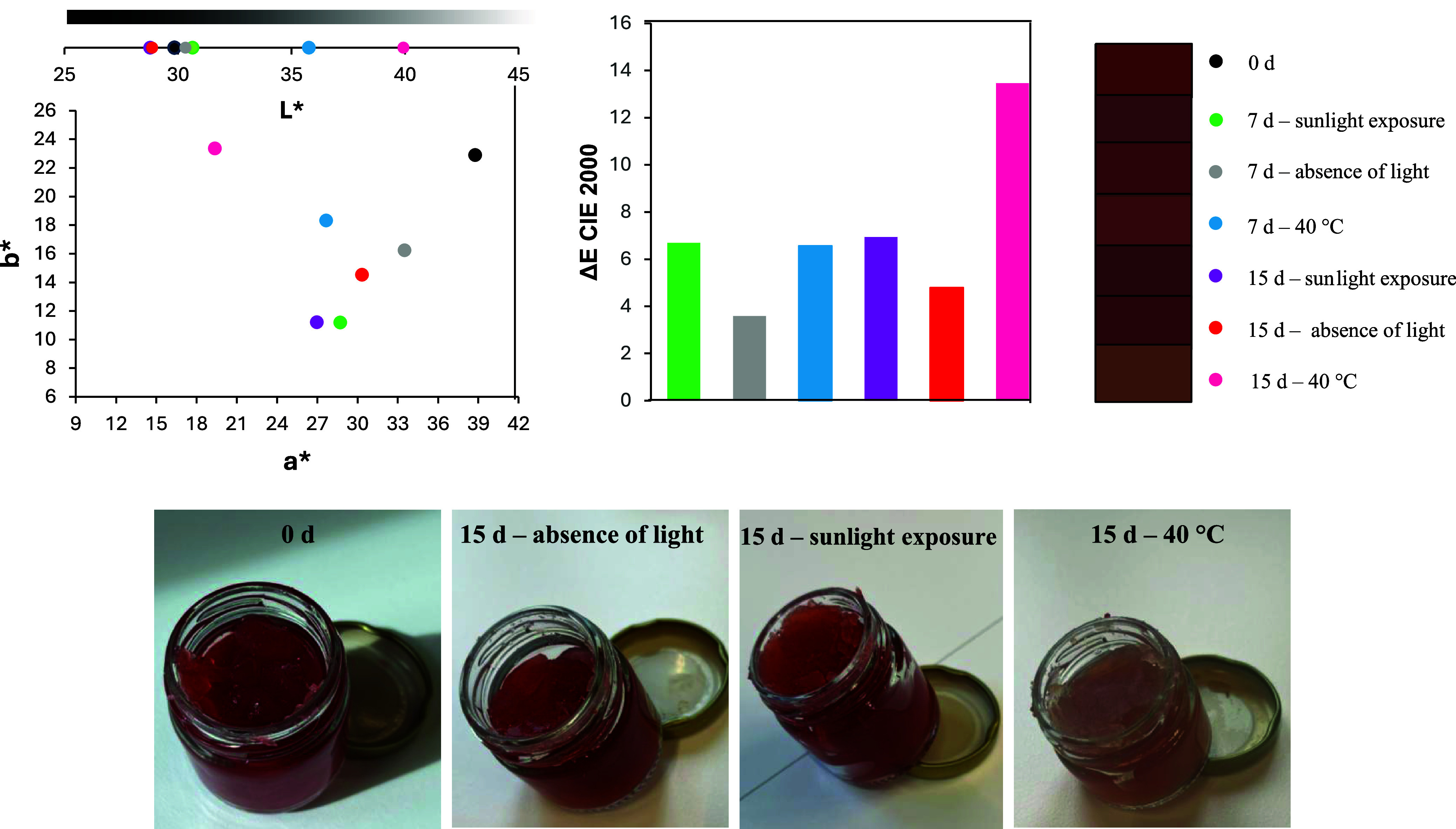
Color properties in the CIELAB system of the multifunctional jelly stick formulation with 1 wt % of lyophilized extract under different storage conditions over time.

### 3.7 Matrix SWOT of Natural Colorants Used in Cosmetic Formulations

The SWOT analysis provides a structured comparison between natural red colorants (for example, azaphilones) and synthetic colorants, namely Red Dye No 3 (erythrosine) and Red Dye No 40 (Allura Red AC), highlighting critical factors influencing their adoption in cosmetic formulations. Below, we discuss the implications of each category (Strengths, Weaknesses, Opportunities, Threats), as presented in Figure 7.

**Figure 7:**
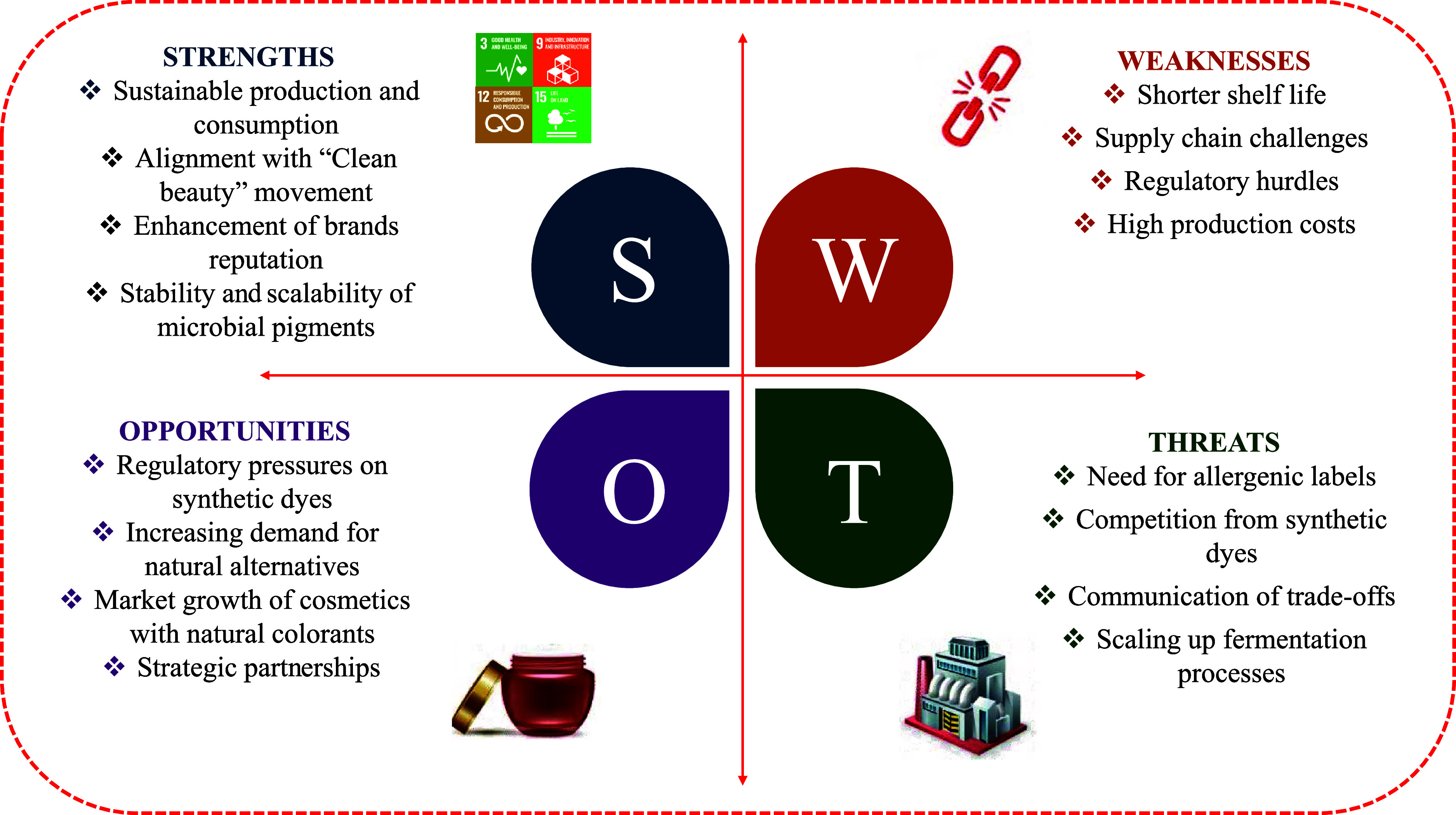
SWOT analysis to evaluate the potential of natural red colorants as alternatives in cosmetic formulations.

#### 3.7.1 Strengths (S)

Natural red colorants are increasingly favored for their plant- or microbial-based origins, aligning with the “clean beauty” movement and consumer demand for safer, sustainable ingredients.^34^ Unlike synthetic colorants, such as Red Dye No. 40, which have been linked to allergic reactions and behavioral concerns in children,^35^ and Red Dye No. 3, which are associated with renal and thyroid disorders and some types of cancer.^36^ In general, natural colorants enhance brand reputation by catering to eco-conscious consumers. Thus, brands using fruit-derived colorants can leverage “upcycling” narratives, while those using microbial alternatives might emphasize carbon-neutral production or non-GMO fermentation. Furthermore, in alignment with the UN’s 2030 Agenda, microbial colorants advance SDG 12 (Responsible Consumption and Production) by enabling more sustainable manufacturing. Their bio-based nature also contributes to climate action (SDG 13) and life on land (SDG 15), offering a sharp contrast to petrochemical-based synthetics like Red Dye No. 40. Additionally, microbial colorants often exhibit superior stability to plant-based alternatives, including high yield scalability, fermentation-based production (reducing agricultural land use), and consistent quality control compared to plant extracts.

#### 3.7.2 Weakness (W)

Despite their safety and sustainability advantages, natural colorants face significant challenges that limit their widespread adoption in cosmetics. Natural colorants often degrade under UV light, heat, or pH fluctuations, leading to shorter shelf lives and inconsistent performance compared to synthetic dyes,^37^ requiring compensation in the development of cosmetic formulations to improve their stability.^38^ Additionally, natural colorants suffer from supply chain challenges, such as high production costs due to low extraction yields and batch inconsistency. Another challenge is related to regulations, In the EU for example, where synthetic dyes face strict scrutiny, natural alternatives are not exempt from regulatory hurdles; some may even be deemed unsuitable for use due to undefined safety thresholds, lack of standardized testing protocols, or restrictions on certain raw materials.^39^

#### 3.7.3 Opportunities (O)

Growing regulatory pressures, such as bans on synthetic colorants in schools and stringent regulatory agencies’ labeling requirements are driving demand for natural alternatives. The FDA’s inclusion of certain plant- and microbial-based reds on its GRAS list further signals a shift toward safer, sustainable ingredients.^40^ This trend aligns with the projected 5.3% annual growth of the global natural cosmetics market (2024–2030), fueled by consumer preference for clean-label products and aversion to synthetic additives.^41^

Successful brands have demonstrated the market potential of natural colorants, containing those produced by microorganisms, such as the red ones, with a distinct alternative, including superior scalability, fermentation-based production, and independence from seasonal crop cycles. Strategic partnerships with agricultural suppliers could optimize substrate sourcing for microbial fermentation, while quality certifications may enhance consumer trust. By capitalizing on these opportunities, microbial colorants may position themselves as a viable, future-proof solution, combining regulatory compliance, environmental sustainability, and economic viability of cosmetic formulations.

#### 3.7.4 Threats (T)

Natural colorants face complex approval processes by requiring allergenic labels, discouraging use despite their efficacy. Novel microbial colorants (for example, from *Talaromyces*) may face lengthy EU Commission evaluations. Synthetic dyes mimic natural hues with better stability, blurring the clean beauty narrative. This is why Red Dye No 40 remains dominant in mass-market cosmetics due to its affordability and performance. Natural colorants must compete on both functionality and ethics, requiring transparent communication of trade-offs (for example, shorter shelf life vs safety benefits). Despite their potential, Sharma et al.,^42^ described that microbial colorants face significant threats, including high production costs and technical challenges in scaling up fermentation processes to industrial levels, which could hinder commercialization and cost competitiveness compared to conventional synthetic colorants.

## 4 Conclusion

This study demonstrated that natural red lyophilized extract may be effectively used in cosmetic formulations as an alternative to synthetic colorants. The microbial colorant showed strong antioxidant activity and low cytotoxicity at concentrations suitable for topical use, indicating its safety for cosmetic applications. Cosmetic formulations were successfully developed with 1 and 5 wt % of the extract. These formulations had appropriate pH levels and maintained good rheological and textural properties. The addition of the colorant did not negatively affect texture, and in some cases, improved spreadability and reduced stickiness.

Among the tested concentrations, 1 wt % was selected as ideal for further studies due to its balance between performance and cost. Stability tests showed that the color of the formulations may change over time, especially when exposed to high temperatures. Thus, the results presented in this work show microbial colorants as a suitable alternative for cosmetics formulations, offering functional and environmental advantages over conventional synthetic colorants.

## Supplementary Material



## Data Availability

Not applicable.

## Notes

Table S1—Quantitative formulation of facial creams; Table S2—Quantitative formulation of shampoo; Table S3—Quantitative formulation of multifunctional jelly sticks. Figure S1. The relationship between shear rate and shear stress for cosmetic formulations with added colorant: (A) Shampoo; (B) Face cream (PDF)
