# Supplementary material for: Redefining Red: Microbial Polyketides in Eco-Friendly Cosmetic Development
Source: ACS Omega. 2025 Dec 8;10(50):62300–11. doi: 10.1021/acsomega.5c10255 (PMC12750396; doi:10.1021/acsomega.5c10255)
Supplement: Supplementary file 1 [file ao5c10255_si_001.pdf]

Supplementary Material

Redefining Red: Microbial Polyketides in Eco-Friendly Cosmetic Development

Juliana Barone Teixeira[a], Pedro Garcia Pereira Silva[a], Júlio Gabriel Oliveira de Lima[a], Matilde Carvalho[b], Lídia M. Gonçalves[b], Nathalia Vieira Porphirio Veríssimo[c], Joana Marques Marto[b,*], Valéria C. Santos-Ebinuma[a,*]

[a] Department of Bioprocess Engineering and Biotechnology, School of Pharmaceutical Sciences, São Paulo State University, 14801902, Araraquara, SP, Brazil.

[b] Research Institute for Medicine (iMed, Ulisboa), Faculty of Pharmacy, University of Lisbon, Lisbon, Portugal.

[c]Department of Pharmaceutical Sciences, School of Pharmaceutical Sciences of Ribeirão Preto (FCFRP), University of São Paulo (USP), Ribeirão Preto, Brazil.

ORCID:

Juliana Barone Teixeira: 0009-0001-6199-66870009-0001-6199-6687

Pedro Garcia Pereira Silva: 0000-0002-2623-9744

Júlio Gabriel Oliveira de Lima: 0000-0001-7403-5191

Matilde Carvalho: 0009-0005-1454-3414

Lídia M. Gonçalves: 0000-0002-6799-2740

Nathalia Vieira Porphirio Veríssimo: 0000-0001-9978-6864

Joana Marques Marto: 0000-0001-5523-5622

Valéria de Carvalho Santos-Ebinuma: 0000-0002-6666-6695

*Corresponding author: Joana Marques Marto - E-mail: jmmarto@ff.ulisboa.pt, Valéria C. Santos-Ebinuma - E-mail: valeria.ebinuma@unesp.br

Number of Pages: 3

Number of Tables: 3

Number of Figures: 1

Table S1 – Quantitative formulation of facial creams.

Disperse Phase	Ingredient (INCI)	Formulations (wt%)		
		Contro l	F1	F5
Oily Phase	Polyglyceryl – 3 Dicitrate/stearate	3	3	3
	Diethylhexyl carbonate	2.5	2.5	2.5
	Glyceryl stearate	0.5	0.5	0.5
	Cetearyl alcohol	2.5	2.5	2.5
	Olea Europaea fruit oil	2.5	2.5	2.5
	Shea butter	2.5	2.5	2.5
	Caprylic/capric triglyceride	5	5	5
	Tocopherol	0.5	0.5	0.5
Aqueous Phase	Glycerin	5	5	5
	Phenoxyethanol	1	1	1
	Azaphilone polyketides extract	-	1	5
	Aqua	75	74	73

Table S2 – Quantitative formulation of shampoo.

Ingredient (INCI)	Formulation (wt%)		
	Control	F1	F5
Sodium cocoamphoacetate	8	8	8
Caprylyl/capryl glucoside	8	8	8
Disodium PEG-5 laurylcitrate sulfosuccinate	8	8	8
Cocamidopropyl betaine	8	8	8
Sodium cocoyl apple amino acids	5	5	5
Glycol distearate (and) steareth-4	3	3	3
PEG-120 methyl glucose dioleate	4.5	4.5	4.5
PEG-7 glyceryl cocoate	3	3	3
Glycerin	2	2	2
Phenoxyethanol	1	1	1
Polyquaternium-7	0.5	0.5	0.5
Azaphilone polyketides extract	-	1	5
Aqua	49	48	47

Table S3 – Quantitative formulation of multifunctional jelly sticks.

Ingredient (INCI)	Formulation (wt%)		
	**Control**	**C1**	**C5**
Aqua	88	87	83
Glycerin	5	5	5
Agar-agar	7	7	7
Azaphilone polyketides extract	-	1	5
Stabileze QM™	-	-	-

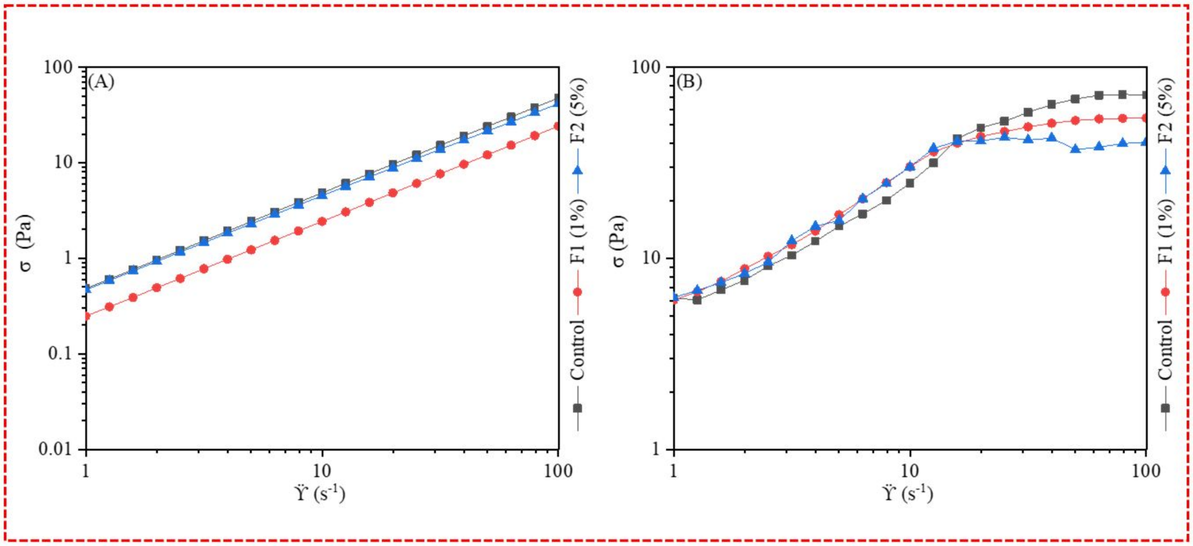

Figure S1. The relationship between shear rate and shear stress for cosmetic formulations with added colorant: (A) Shampoo; (B) Face cream.
